# Information Indicators of Occurrence and Monitoring of Material Structure Degradation in Vibrodiagnostic Systems During Loading

**DOI:** 10.3390/ma18245507

**Published:** 2025-12-08

**Authors:** Artem Sharko, Dmitro Stepanchikov, Oleksandr Sharko, Andriy Buketov, Petr Louda, Bogdan Maslyiak, Valerii Kyrylovych, Vyacheslav Svyrydov, Piotr Czarnywojtek, Marek Dębczyński, Piotr Łoś, Katarzyna Ewa Łoś

**Affiliations:** 1 Institute of New Technologies and Applied Informatics, Faculty of Mechatronics, Informatics and Interdisciplinary Studies, Technical University of Liberec, Studentská 1402/2, 46117 Liberec, Czech Republic; 2 Department of Energetics, Electrical Engineering, and Physics, Kherson National Technical University, 73008 Kherson, Ukraine; dmitro_step75@ukr.net; 3 Department of Transport Technologies and Ship Repair, Kherson State Maritime Academy, 73000 Kherson, Ukraine; avssharko@gmail.com (O.S.); buketov@tntu.edu.ua (A.B.); 4 Polytechnic Faculty, University of Kalisz, Pl. Wojciecha Bogusławskiego 2, 62-800 Kalisz, Poland; petrlbc1@seznam.cz (P.L.); p.czarnywojtek@uniwersytetkaliski.edu.pl (P.C.); m.debczynski@uniwersytetkaliski.edu.pl (M.D.); 5 Department of Specialized Computer Systems, Faculty of Computer Information Technologies, West Ukrainian National University, 11 Lvivskast., 46009 Ternopil, Ukraine; bm@wunu.edu.ua; 6 Department of Robotics, Electric Power Engineering and Automation named after Prof. B.B. Samotokin, Faculty of Computer-Integrated Technologies, Mechatronics and Robotics, Zhytomyr Polytechnic State University, Chudnivska St., 103, 10005 Zhytomyr, Ukraine; kiril_va@yahoo.com; 7 Department of Ship Engineering and Power Engineering, Kherson Educational-Scientific Institute of the Admiral Makarov National University of Shipbuilding, Nezalezhnosti Ave., 44, 73003 Kherson, Ukraine; viacheslav.svyrydov@nuos.edu.ua; 8 Department of Machine Parts and Mechanism, Faculty of Mechanical Engineering, Technical University of Liberec, Studentská 1402/2, 46117 Liberec, Czech Republic; piozyt@gmail.com

**Keywords:** vibration diagnostics, degradation, information indicators, plain bearings, marine vehicles, material structure

## Abstract

A methodology of statistical processing has been developed and practical implementations of the application of vibration signals in the analysis of the evolution of damage accumulation in ship bearings have been performed. It has been shown that vibration signals are multicomponent, representing a finite additive set of multi-scale components localized in time and frequency domains of different vibration types. A conceptual model and algorithm for searching for optimal information indicators in technical condition monitoring systems, based on reducing the dimensionality of input information using the principal component method, have been developed. In this paper, the principal component method is approximated by an n-dimensional observation region in an n-dimensional ellipsoid, the semiaxes of which will be the main components. In this case, the input data matrix is transformed into a matrix of normalized centered values, and the set of points is represented by their distances to straight lines and planes. The tangent to the exponential trend of change in the corresponding component in the pre-destruction area has been chosen as the criteria for assessing the approach to the state of degradation and failure. It is shown that among the studied components, the first and third components are the most informative. The specification of the main components reflects the linear diversity of statistical features of vibration signals and can be an indicator of the state of the object under study. Thanks to vibration analysis, the user receives information about the technical condition and approach to degradation of the material.

## 1. Introduction

Potential opportunities for obtaining reliable and up-to-date information about the operational processes of judicial transport vehicles in digital form. The digitization of technical diagnostics is based on complex interrelationships between information and communication elements, which are based on the monitoring and processing of a large array of experimental data in the digital space. This makes it possible to significantly reduce the number of field tests, select the optimal strategy, track digital trends, and find technological solutions for searching for new diagnostic information parameters. Studying digital trends and using them in diagnostics is the basis for preventive measures and warnings about impending danger.

Among the existing and practical methods for assessing the reduction in the strength properties of materials under conditions of uncertainty due to external influences, the most common is the vibroacoustic diagnostics method, based on the analysis of the spectral characteristics of equipment during operation.

Vibration diagnostics involves the detection and identification of defects by identifying characteristic vibration patterns, determining the type and depth of defects, and predicting their development.

Vibration diagnostics involves measuring vibrations in the low and medium frequency range at points on the body that are distant from vibration-active components.

The uncertainty of the transport sector, extreme and peak loads, deviations from normal operating conditions, as well as sharp fluctuations in weather and navigation conditions when passing complex routes determine the magnitude of loads on marine plain bearings. The search for optimal information indicators of vibration diagnostics in systems for monitoring the technical condition of marine plain bearings determines the assessment of their condition during operation. The development of diagnostic systems is impossible without the use of information and computing tools, the software of which is based on fundamental and applied research.

If metallurgical defects can be detected by flaw detection and non-destructive testing, then to detect operational defects, a whole range of measurements of their indirect manifestations is required.

The acute practical relevance of the research topics determines the urgency of finding new methods for determining the degradation of material properties during extreme fluctuations in technological and operational factors.

The technical state of plain bearings can be characterized by a large number of diagnostic parameters, including defectoscopy parameters of the selected unit, instrumental wear parameters, analysis of the physical and chemical properties of the used lubricant, tribocoupling, as well as electrical, thermal, and vibroacoustic characteristics. Interpretation of the results of such complex measurements is difficult due to possible discrepancies in the conclusions of the methods used, and sometimes their internal inconsistency. Therefore, the development of new methods for assessing the technical state of marine plain bearings is relevant and timely.

## 2. Statement of the Problem

Many years of practical experience in the operation of plain bearings of marine power plants shows that monitoring the parameters of vibration diagnostics is one of the main information tools for assessing their technical condition.

Vibration methods for diagnostics and assessing the technical state of plain bearings, including their service life, are currently the most effective ways to ensure marine transport equipment’s reliability and trouble-free operation.

The physical processes occurring in plain bearings during their operation are quite complex and depend on the design features of the bearing and the ratio of many external and internal factors that determine the operating conditions. The most effective method for studying vibration properties is spectral analysis. When significant changes occur in the shape of the spectrum surfaces due to rotor wear, low-frequency vibration components arise. Diagnostics of plain bearings by low-frequency vibration makes it possible to detect defects in places that are difficult to reach for measurements. Diagnostics of plain bearings by high-frequency vibration makes it possible to detect defects associated with the operating features of bearings, including the entire bearing unit. Therefore, the list of defects detected during diagnostics of plain bearings includes not only those that relate to the bearing itself, but also those that relate to violations of its operation. All these defects together affect the service life of the bearing.

The accuracy of diagnostics directly depends on the quality of the input information. This is ensured by the use of digital technologies. The creation of digital platforms is intended for collecting and processing data in digital format, establishing digital trends of diagnostic parameters. Vibration diagnostics uses a large number of diagnostic parameters, the statistical characteristics of which change in the time and frequency domains during post-processing.

The main resource for ensuring the intended guidelines is information. Lack of information does not allow for making adequate decisions, and redundancy complicates its use and creates difficulties in identifying the useful component. The search for new diagnostic parameters and information indicators of the technical condition of rotor mechanisms of marine power plants, which includes plain bearings, is not only a scientific task but also an urgent technical one.

The purpose of the work is to find optimal information indicators of vibration diagnostics in monitoring systems for the technical condition of marine plain bearings.

## 3. Analysis of the Latest Achievements and Publications on the Issue

The potential of vibroacoustic methods and their prospects as a means of diagnosing the strength properties of materials require efforts to be focused on improving methods and information characteristics of vibration signals that indicate the onset of degradation.

In [1,2,3], the results of diagnostics based on the analysis of the envelope of vibration accelerations generated by the valve mechanism and the fuel system of high-speed marine diesel engines of Kobben-class submarines are presented. Engines of this type do not have indicator valves, which complicates the assessment of their technical condition. In [4], the results of studies on the influence of the operation of a marine diesel engine on the vibroacoustic characteristics of real operating conditions are described.

In [5,6,7], it is proposed that the area covered by the vibration signal characteristic be used as a criterion for the effectiveness of bearing diagnostic parameters. Diagnostic parameters of vibration signals in their multimode measurement for rotating mechanisms are presented in [8]. In [9], it is proposed to determine the presence of equipment malfunctions by changing the trend of vibration signals. The problem of improving the quality of vibration diagnostics of bearings of rotating mechanisms is constantly in the center of attention of research in its various directions and new diagnostic parameters. Difficulties in implementation associated with unbalanced data samples in long time series and optimization of the distribution of features of two-dimensional maps are noted.

The application of the principal component method of vibration diagnostic signals to assess the condition of bearings is described in [10]. The need to specify the statistical characteristics of vibration signals for various monitoring purposes is shown. Multifunctional navigation systems are presented in the vessel’s parametric rolling on the wave [11], and in case of inevitable collision of ships [12], innovative transformations resources management in [13], intelligent systems [14], and entropy models [15] work in determining whether the residual resources are presented in [16] diagnostics of rolling bearings and monitoring of plain bearings [17]. For multi-criteria assessments [18] and determination of geopolymer concentrations [19,20,21].

Based on the analysis of the latest achievements in the field of vibration diagnostics of rotating equipment, development trends and critical gaps in research have been identified, such as the need for adaptive algorithms for real-time signal processing, the search for optimal information indicators for vibration diagnostics of plain bearings, as well as the development of diagnostic and monitoring schemes for observing material degradation during operation under difficult loading conditions.

## 4. Materials and Methods

Diagnostic signals from monitoring of TPL-88 marine plain bearings with a maximum shaft speed of 12,000 rpm were selected as research materials.

**Figure 1:**
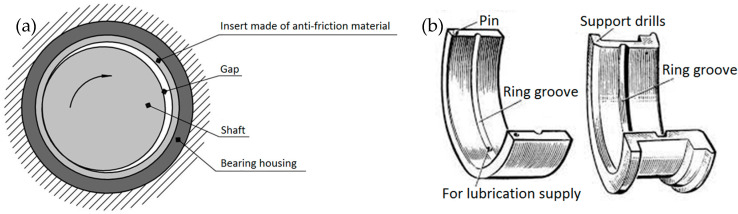
Design of a plain bearing: (**a**) operating principle, (**b**) design features.

In terms of design, plain bearings consist of a housing, an antifriction liner, a part of the shaft surface, a bearing journal, and a layer of oil between them (Figure 1).

According to their purpose, plain bearings are divided into thrust and support bearings. Support bearings limit radial movements and thrust bearings limit axial movements. On modern ships, liners are installed in pairs, which are fixed from rotation by special cylindrical pins.

**Figure 2:**
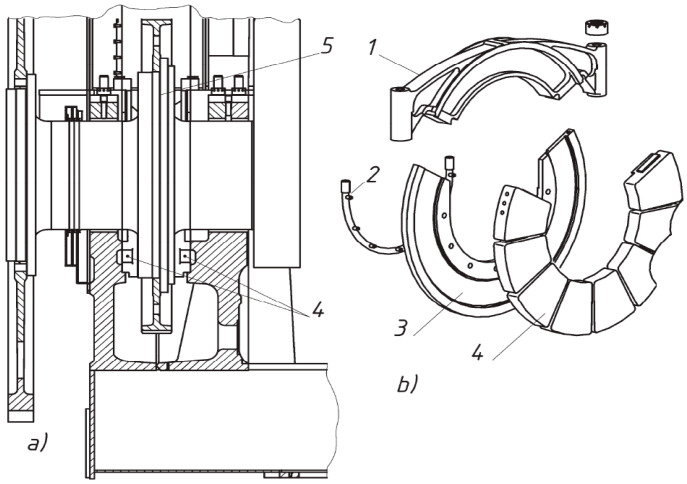
Thrust bearing unit for low-speed diesel engines of the X72 series by WinGD (Winterthur, Switzerland) (**a**) and the K, L, S series by MAN (Munich, Germany) (**b**); 1—upper cover; 2—lubricating oil supply tube; 3—thrust plate; 4—thrust segment; 5—thrust comp.

For a more uniform supply of oil to the entire surface of the bearing, the surface of the liners has annular oil distribution grooves, thinnings, and oil pockets to remove wear products from the friction zone (Figure 2).

The oil film is formed due to the hydrodynamic effect. When the shaft rotates, the lubricant is drawn into the gap and distributed evenly. The most common types of friction bearing defects are as follows:

- Wear of the inner surfaces of bushings and liners;
- Scoring on sliding surfaces;
- Wear of the ends of liners;
- Failure of liners and bushing fasteners.

The vibroacoustic method of assessing the quality of materials with processing of diagnostic signals by the principal component method was selected as the research method. The novelty and originality of the proposed technique lie not so much in measuring vibration signals at different times of operation of plain bearings, but in creating promising methods for processing diagnostic signals. The variety of statistical indicators does not allow us to unambiguously determine the trends in the development of defects and diagnose them, since in addition to quantitative estimates of indicators characterized by different measurement scales, there are also their qualitative differences. Presentation of experimental information and its processing by the principal component method allows combining the influence of factors of different dimensions, compressing dimensions, as well as visualizing and interpreting measurement results. The principal component method transforms multidimensional data, projecting them onto a space with fewer dimensions. In this case, noise is removed, focusing on basic patterns or trends occurring. The method reduces the number of variables in the data set, preserving the most important information and reduces its computational complexity.

In vibration diagnostics, 90–95% of information about the technical condition of a machine is contained in just 2–5 main components, which significantly reduces the amount of data analyzed without losing key information.

## 5. Methodology

The principal component method allows combining the diversity of different dimensions of statistical features of vibration signals into a single generalizing indicator without losing input information. Technologically, for a set of vectors *x*_1_, *x*_2_, …, *x_m_* ∈ *R^n^*, linear manifolds are values such as *S_k_* ⊂ *R^n^*, in which the sum of the squares of the deviations *x_i_* from *S_k_* will be maximum, i.e.,

(1) $$\sum_{i=1}^{m}dist^{2}(x_{i},S_{k})\to \min$$

where *k* = 0, 1, 2, …, *n* − 1 is the linear manifold in *R^n^*, and *dist* (*x_i_*, *S_k_*) is the Euclidean distance

Calculation of the principal components is reduced to the singular decomposition of the eigenvectors of the covariance matrix of the original data. Formalization of the use of the principal component method is the construction of an orthogonal transformation of coordinates, as a result of which the correlations between individual coordinates will vanish.

Technically, at each step 2*k* − 1, projections are subtracted onto the previous principal component. The vectors found are orthonormalized as a result of solving the optimization problem.

The mathematical content of the principal component method is reduced to the spectral decomposition of the covariance matrix. The implementation of the methodology is associated with continuous periodic recording of control parameters.

Linear manifolds are defined by a set of principal components, vectors {*a*_1_…*a*_K−1_} and vectors *a*_0_, which are determined by minimization *S*_0_.

(2) $$a_{0}=\underset{a_{0}\in R^{n}}{\arg}\left(\sum_{i=1}^{m}dist^{2}\left(x_{i},S_{0}\right)\right)=\underset{a_{0}\in R^{n}}{\arg\min}\left(\sum_{i=1}^{m}{\left|x_{i}-a_{0}\right|}^{2}\right)$$

The variational definition of the mean as the point that minimizes the sum of squares is

(3) $$a_{0}=\frac{1}{m}\sum_{i=1}^{m}x_{i}^{2}$$

The technology for using the principal component method is standard and is therefore described only fragmentarily in this paper. The novelty of this work lies in its adaptation and interpretation of the results obtained.

When projecting onto these axes, the greatest amount of information is preserved. The first principal component maximizes the sample variance of the data projection. It is necessary to find such an orthogonal transformation into a new coordinate system for which the sample variance of the data along the first coordinate is maximum.

The second principal component, provided that the first coordinate is orthogonal, maximizes the sample variance of the data along the second coordinate.

The *k*-th principal component, provided that the *k* − 1 coordinate is orthogonal, maximizes the sample variance of the data along the values of the *k* − 1 coordinate. The solution to the best approximation problem gives the same set of principal components as the search for orthogonal projections with the greatest dispersion. The problems of determining the principal components are reduced in their methodological terms to the problem of diagonalizing a sample of a covariance matrix.

## 6. Experiment

Experimental vibration diagnostics equipment includes sequentially connected measuring objects, vibration sensors, a vibrometer, a spectrum analyzer, and a computer.

Signals from sensors can be digitized and recorded for trend analysis. An accelerometer is used to record vibration levels. A vibration signal with a duration of 6 s was obtained daily for 10 consecutive days. A bearing malfunction occurred, which led to its failure.

Any rotating equipment vibrates during operation. For each mechanism, there is a specific set of vibrations that allows for the diagnosis of both the mechanism as a whole and its individual parts. In technical operation, vibration diagnostics consists of regularly measuring the vibration volume by installing sensors, after which measurements are taken at specific frequencies. The data obtained is compared with previous measurements.

**Figure 3:**
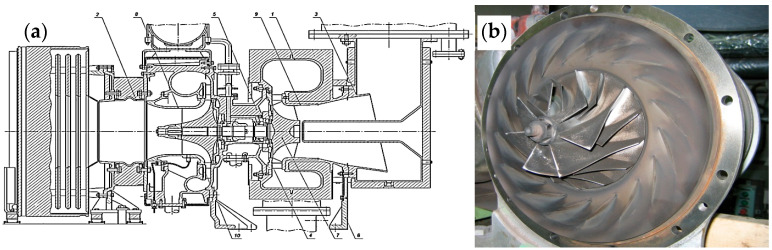
Turbocharger NR34/S102: (**a**) design features; (**b**) operation. 1—compressor housing, 2—air filter, 3—air intake area, 4—sliding bearing, 5—compressor wheel, 6—diffuser, 7—shaft speed sensor, 8—oil accumulator, 9—compressor cleaning device, 10—connecting part of the muffler with the housing.

Measurements are taken on the bearing assembly housing, specifically in its lower part, because this is where the load on the assembly is greatest. The experiment was conducted on friction bearings that are part of turbochargers of four-stroke internal combustion engines. In all four-stroke internal combustion engines, air is compressed using a compressor that is driven by a gas turbine. A turbocharger is a combination of a compressor and a gas turbine (Figure 3).

Turbocharger engine condition indicator: In the structure of all failures, the main reason is the degradation of the parameters and performance characteristics of materials. This can lead to jamming of the rotor shaft due to overheating of the housing, the signs of which are oil leakage from the turbine side. Along with this, there is a strong overheating of the lubricant, its rapid aging, and the formation of deposits on the turbocharger parts. Plain bearings are an important tribological component of the turbocharger. By carrying out diagnostics of plain bearings, it is possible to prevent premature wear and extend the service life of the turbocharger.

Measurements of vibration parameters were carried out on the lower part of the bearing unit, where the loads on the unit are maximum.

The condition of the friction bearing was monitored daily during operation. The results of the observations are presented as a sequence of vibration acceleration pulses over the entire time range up to destruction. To analyze the trends in the change in vibration parameters, they were recorded.

**Figure 4:**
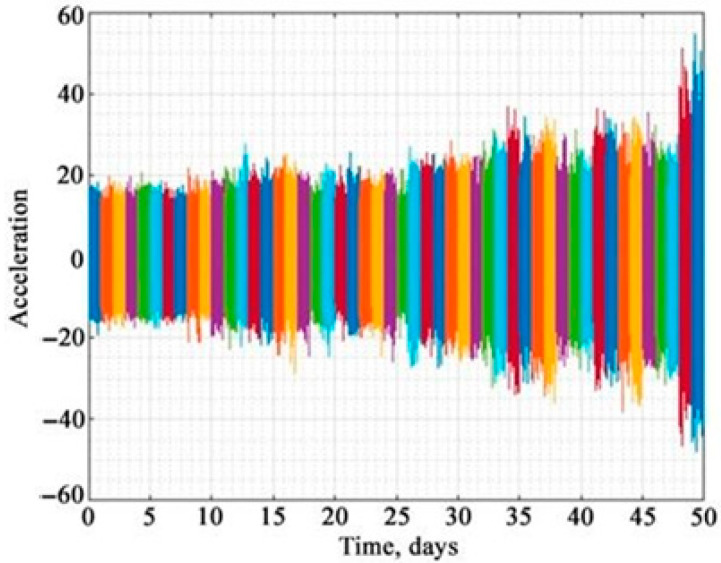
Change in vibration signals during operation of a plain bearing.

Figure 4 shows the set of received vibration signals in the same scale in the order they were recorded.

The colour scheme in Figure 4 is used to visually separate vibration signals by day during the 50-day monitoring process. The vibration signal was received daily for 50 days before the bearing failure. Visual analysis of the vibration parameter trends allows detecting possible changes in the technical condition of the plain bearing. Thus, consideration of Figure 4 shows an increase in the amplitude of oscillations of the analyzed signal as the bearing condition approaches failure. However, the numerical values of the beginning of this process cannot be quantitatively determined and unambiguously established.

In serially produced vibrometers, such spectral characteristics as peak factors, pulse envelopes, their range, standard deviation, and their ratios are used for analysis. To carry out vibration diagnostics of marine plain bearings, in addition to these statistical characteristics of vibration signals and measurements, calculations were also performed for other statistical characteristics in the time and frequency domains of their existence. In the time domain, 11 statistical characteristics were determined: mean value (Mean), standard deviation (Std), skewness, excess (Kurtosis), full swing of oscillations (Peak2Peak), root mean square (RMS), crest factor (CrestFactor), shape factor (ShapeFactor), impulse factor (ImpulseFactor), marginal factor (MarginFactor), and energy (Energy). In the frequency domain, 4 statistical characteristics were determined: mean spectral value (SKMean), standard spectral deviation (SKStd), spectral skewness (SKSkewness), and spectral excess (SKKurtosis). All of the listed statistical characteristics of vibration signals can serve as potential indicators of bearing condition degradation. A filtering and smoothing procedure was applied to these statistical characteristics.

## 7. Results and Discussion

As a result of the work performed, it was established that the trends in the development of diagnostic systems are associated with the development of information and measuring technologies. Diagnostic systems allow the issuing of recommendations for further operation of equipment, continuously monitoring its condition. Modern vibration diagnostics are based on the achievements of many areas of knowledge, which include the following:

- Experience in operating marine power plants;
- Oscillation theory;
- Theory of mechanisms and machines;
- Information theory and modeling.

After measuring vibration signals, they were recorded in the form of digital displays of experimental information, post-processed to identify abnormal bursts in trends, and calculated statistical characteristics of vibration signals at fixed time intervals.

**Figure 5:**
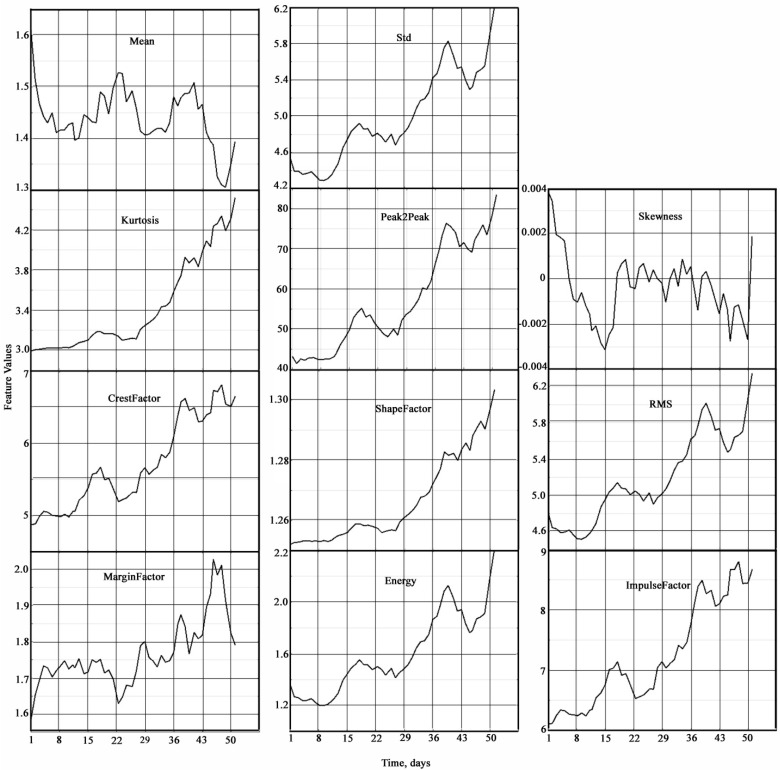
Statistical characteristics of vibration signals of plain bearings in the time domain.

**Figure 6:**
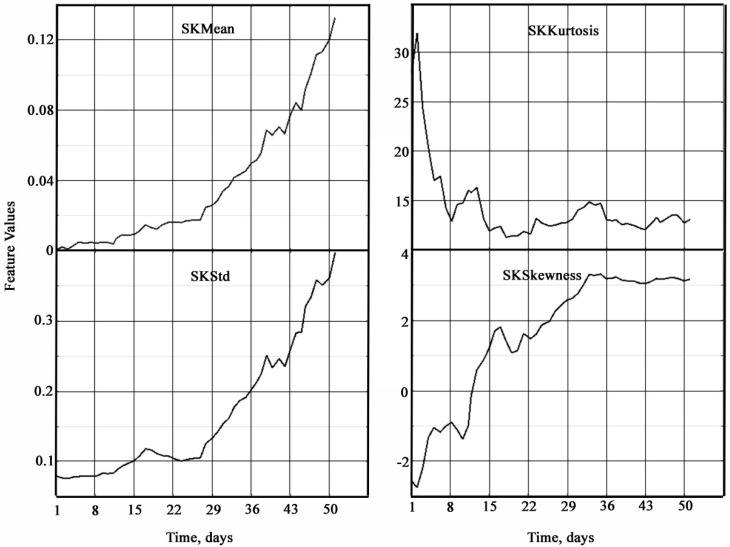
Statistical characteristics of vibration signals of plain bearings in the frequency domain.

All figures and tables in the following description are aimed at identifying degradation conditions and determining its initial stages. In order to clearly present the results of calculations, their comparative visualization was performed on a common time scale with a breakdown of statistical characteristics in the time and frequency domains (Figure 5 and Figure 6).

The temporal dependencies of most statistical characteristics of vibration signals (Figure 5) show an upward trend as the bearing approaches failure. A downward trend is observed for the mean and skewness. The greatest relative change is observed for the peak-to-peak amplitude, skewness, and energy, while the smallest change is observed for the shape factor.

The frequency dependencies of the statistical characteristics of vibration signals (Figure 6) also show significant changes as the bearing approaches failure. Three characteristics—mean value (SKMean), standard deviation (SKStd), and asymmetry (SKSkewness)—show an increasing trend, while excess (SKKurtosis) shows a decreasing trend. A greater relative change is observed in comparison with the characteristics in the time domain. It should also be noted that the oscillatory component of the characteristics in the frequency domain is smaller in comparison with the characteristics in the time domain. These features allow us to conclude that the statistical characteristics of vibration signals in the frequency domain are more sensitive.

The different nature of statistical features of vibration signals in the time and frequency domains and their mathematical interpretation lead to the need to combine them and present them as separate principal components. The numerical values of the first six principal components are given in Table 1.

The nature of the change in the main components can be described in accordance with the physical essence of the material degradation processes occurring during the operation of a plain bearing. The relationship between the main components and the mechanisms of rolling bearing degradation is one of the most powerful applications of the main component method in vibration diagnostics. Principal components act as detectors of hidden faults, as they can automatically extract patterns from a complex vibration spectrum that corresponds to specific defect signatures.

Bearing degradation (e.g., wear, chipping, cracking) develops in stages, and each stage has its own unique vibration signature (Table 2).

**Figure 7:**
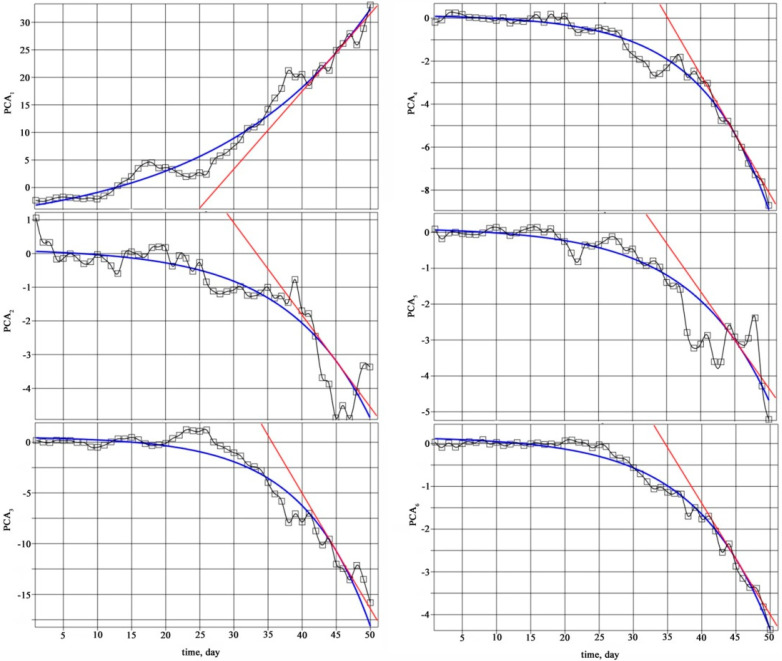
Time dependence of the first six principal components: dots are experimental data, blue line is exponential approximation of the trend, and red line is tangent to the exponential approximation on the 45th day of bearing operation.

The time dependence of the first six components on different days of bearing operation is shown in Figure 7.

The exponential function used in the work has the form

(4) $$PCA_{i}\left(t\right)=a_{i}+c_{i}\exp\left(b_{i}t\right)$$

where

*a_i_*, *b_i_*, *c_i_*—approximation coefficients;
*t*—bearing operating time in days;
*i*—main component number.

An exponential trend in vibration diagnostics means a very sharp and accelerating increase in the vibration level of equipment over a certain period of time. This is one of the most critical trends, as it indicates the rapid development of a serious defect that can lead to sudden machine failure.

Such approximation of statistical features is performed, taking into account the criteria of comparative analysis of information indicators of vibration diagnostics in systems for monitoring the technical condition of marine transport vehicles. The tangent to the exponential trend of change in the main component in the pre-destruction area, the root mean square deviation (RMSE), and the correlation coefficient were used as such criteria (*R*-square).

The main methodological approach to finding optimal information indicators of vibration diagnostics in systems for monitoring the technical condition of marine plain bearings is to assess sensitivity to determining the stages of material degradation.

The sensitivity of the main components to changes in the bearing condition was assessed by the steepness of the change in the characteristics in the pre-destruction area. The steepness is the angular coefficient of the tangent drawn at a certain point of the exponential trend. The 45th day of bearing operation was chosen as such a point, which corresponds to the middle of the pre-destruction area. The tangent equation has the form:

(5) $$\xi_{i}\left(t\right)=PCA_{i}\left(t_{1}\right)+\frac{d}{dt}PCA_{i}\left(t_{1}\right)\left(t-t_{1}\right),$$

where *t*_1_ = 45 days; the slope is determined by the equation

(6) $$k=tg\;\alpha =\frac{d}{dt}PCA_{i}\left(t_{1}\right)=c_{i}b_{i}\exp\left(b_{i}t_{1}\right),$$

where *α* denotes the acute angle between the tangent and the positive direction of the horizontal axis on the graph (Figure 7).

The sensitivity criterion based on the slope of the tangent (derivative) is critical in vibration diagnostics for assessing the current technical condition of equipment and predicting the development of defects. It is applied not directly to the original vibration signal, but to diagnostic parameters or trends in their change. A significant tangent slope is a sign that a defect is actively developing, requiring urgent attention and failure prediction. A negative tangent slope may be a sign of equipment instability.

Since the exponential trend of the first principal component is increasing, and decreases for the remaining principal components, for a correct comparison of the angular coefficients of the tangents, the modulus of the right-hand side of Equation (8) should be taken:

(7) $$k=\left|tg\alpha \right|=\left|c_{i}b_{i}\exp\left(b_{i}t_{1}\right)\right|,$$

Accordingly, for the angle *α*, we have

(8) $$\alpha =\left|arctg\left(c_{i}b_{i}\exp\left(b_{i}t_{1}\right)\right)\right|,$$

Greater sensitivity corresponds to a greater steepness of the tangent, i.e., a greater value of the angular coefficient *k* and the angle *α*.

The numerical values of the approximation coefficients, approximation errors, as well as the angular coefficients and slope angles of the tangents for the first six principal components are given in Table 3.

The errors in approximating the trends of the main components in Table 2 are presented by calculating the root mean square error (RMSE) and the correlation coefficient (R-square). In this case, the characteristic with the smallest root mean square error and the largest correlation coefficient should be considered the best for use as a diagnostic.

Sensitivity in Table 2 is described by the angular coefficient *k* and the angle of inclination *α* of the tangent. The characteristic with the highest values of *k* and *α* is considered the best for diagnostic use.

In our final recommendation, we considered the sensitivity of the main component to defect development to be the most important criterion.

The analysis of the data in Table 2 shows that the first principal component PCA1 has the highest sensitivity and the highest correlation coefficient. The third principal component PCA3 is in second place according to these criteria. All other principal components considered have low sensitivity. Thus, the conducted study allows us to recommend the first and third principal components for vibration monitoring.

The conclusions obtained are quantitatively confirmed by the results of measurements and calculations based on their processing.

**Figure 8:**
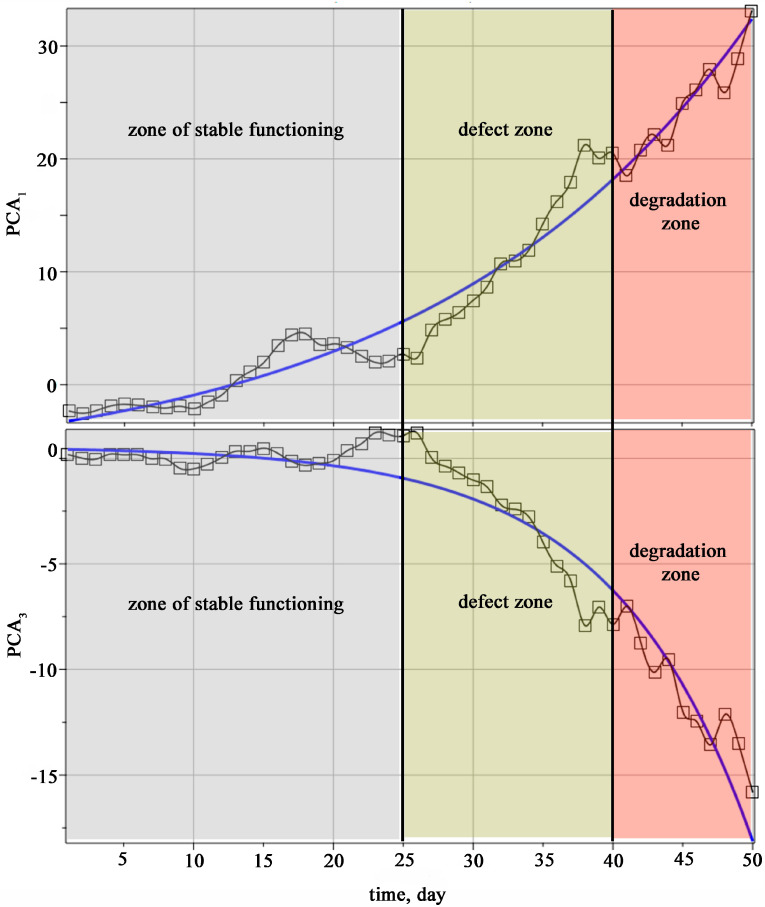
Areas of material defect development during different periods of sliding bearing operation, determined by recommended diagnostic indicators.

**Figure 9:**
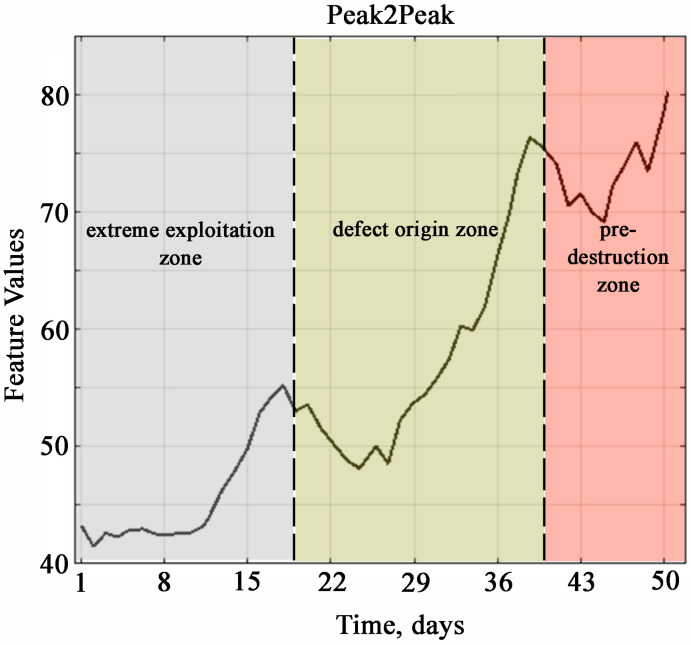
Areas of material defect development during various periods of sliding bearing operation.

Figure 8 and Figure 9 show the zones of development of material defects in different periods of operation of plain bearings under conditions of uncertainty of operational loads.

Areas of material defect development during various periods of sliding bearing operation, determined by the characteristic of the full swing of oscillations, which has the greatest weight in the first principal component. Figure 8 and Figure 9 clearly show the characteristic zones of performance of plain bearings during operation up to the destruction of the material. The zone of stable functioning of the diagnostic object, the zone of defect initiation, and the degradation zone are defined.

The results presented in this paper are based on the monotonicity of the measured characteristics, their quantitative shifts, and the possibility of forecasting degradation.

**Figure 10:**
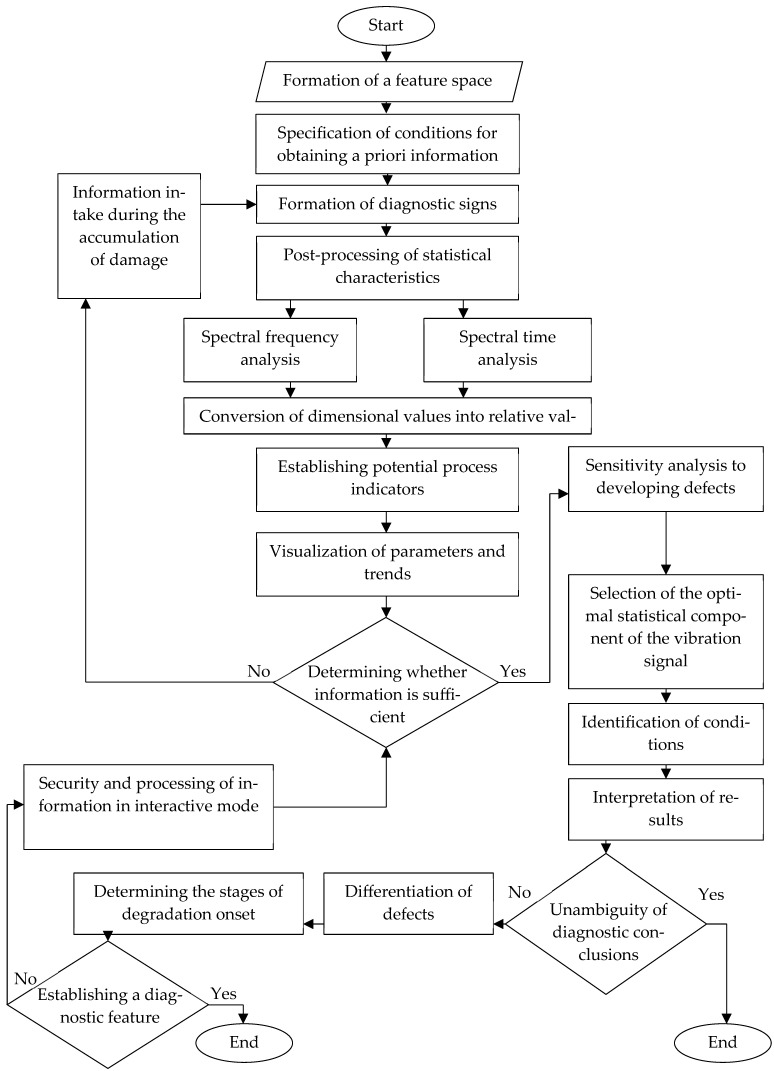
Algorithm for determining diagnostic signs of material degradation.

An algorithm has been developed for comparative analysis of vibrodiagnostic information indicators in ship technical condition monitoring systems, based on establishing sensitivity to determining material degradation (Figure 10).

When constructing the algorithm, the possibilities of describing the interactive interaction of users with the processes of assessing the technical condition of plain bearings and determining the stages of material degradation in a dialog mode by comparing the sensitivity trajectory of the diagnostic feature with the zone of identification of degradation and operability of the bearing were taken into account. The novelty of the proposed algorithm is the elimination of the need to compare the results of vibration diagnostics of the controlled and reference bearings, since it is impossible to ensure their functioning under identical operating conditions. Thanks to the vibration analysis, the user receives information about the technical condition of the object and the approach to material degradation.

The authors have previously presented the problem of monitoring the condition of marine bearings in several papers.

The main purpose of monitoring is to observe changes in diagnostic information obtained during the assessment of the technical condition of marine plain bearings, characterizing the onset of material degradation.

Integration of vibration monitoring of marine plain bearings into the local network of equipment safety and reliability allows for data collection and analysis with the issuance of recommendations in any convenient location or to configure the diagnostic process for autonomous operation.

## 8. Conclusions

1. Ensuring the reliability and failure-free operation of marine plain bearings requires mandatory diagnostics and monitoring of their technical condition during operation. Among the most methods of technical diagnostics, the most promising is vibration, based on the transformation of material structure degradation into a diagnostic signal. The patterns of change in vibroacoustic features are determined by the patterns of structural damage and material degradation under loading.
2. It is proposed to use the principal component method in pre-processing the statistical characteristics of vibration signals to determine new diagnostic features and reduce the dimensionality of input information. The calculations of the first six principal components showed that the nature of their change can be described in accordance with the physical essence of material degradation processes during bearing operation. The use of these principal components allows observing the stages of defect development in real time.
3. It is proposed to estimate the sensitivity of the principal components to changes in the state of plain bearings by the steepness of the change in the component characteristics in the pre-destruction area. Calculations of approximation coefficients, errors, and sensitivity parameters for the first six principal components showed that the first and third principal components can be recommended as diagnostic features for vibration monitoring of the technical condition of marine plain bearings. Using the growth rate of these diagnostic features as a new information parameter that determines the sensitivity of the method to establishing the initial stage of damage accumulation is a fundamentally new direction in vibration diagnostics.

## Figures and Tables

**Table 1:** Numerical values of the first six principal components.

Day	PCA_1_	PCA_2_	PCA_3_	PCA_4_	PCA_5_	PCA_6_
1	−2.32196	1.056003	0.180322	−0.18943	0.094573	0.037472
2	−2.52862	0.332577	0.014825	−0.07762	−0.1812	−0.08838
3	−2.27081	0.326488	−0.03791	0.247356	−0.01774	0.012157
4	−1.87704	−0.17082	0.200749	0.253957	0.004032	−0.08354
5	−1.73585	−0.14708	0.166487	0.177332	−0.03695	0.00027
6	−1.82545	−0.00791	0.171883	0.043549	−0.05678	0.045118
7	−1.94189	−0.13279	−0.0193	0.025289	−0.06397	0.023805
8	−2.06353	−0.30587	−0.02302	−0.01136	−0.01305	0.095327
9	−1.90792	−0.24795	−0.46913	−0.03618	0.112105	−0.01204
10	−2.10126	−0.03216	−0.4939	−0.10205	0.140691	0.029246
11	−1.5351	−0.16109	−0.29458	0.026909	0.056826	−0.047
12	−0.8902	−0.36384	0.034585	−0.22863	−0.08673	−0.01216
13	0.340537	−0.59541	0.334295	−0.12534	−0.01876	0.024671
14	1.153248	−0.00997	0.33278	−0.14998	0.071634	−0.04551
15	1.967237	0.048682	0.483187	−0.02672	0.129731	−0.00351
16	3.463859	−0.03487	0.249752	0.156461	0.140884	0.018294
17	4.377934	−0.10398	−0.14131	−0.18876	0.007945	−0.01777
18	4.522902	0.176948	−0.33646	0.191686	0.102205	−0.00772
19	3.549559	0.196337	−0.23948	−0.08262	−0.13174	−0.03568
20	3.624352	0.176692	−0.11378	0.096152	−0.25372	0.066944
21	3.272693	−0.37611	0.37252	−0.35391	−0.5738	0.086013
22	2.558962	−0.05943	0.690423	−0.66467	−0.82212	0.030802
23	1.970163	−0.14109	1.221505	−0.53177	−0.3526	0.01036
24	2.092096	−0.52966	1.124865	−0.59729	−0.38516	−0.09749
25	2.697314	−0.26672	1.078035	−0.44893	−0.3414	−0.025
26	2.384266	−0.84328	1.199066	−0.51762	−0.22655	−0.0704
27	4.813481	−1.11717	0.027569	−0.6057	−0.11561	−0.26716
28	5.758266	−1.20307	−0.36869	−0.69798	−0.25992	−0.34062
29	6.376734	−1.12879	−0.6967	−1.3296	−0.50683	−0.38207
30	7.470175	−1.0821	−1.03646	−1.6893	−0.46219	−0.56225
31	8.668388	−0.98006	−1.35455	−1.91379	−0.78781	−0.70556
32	10.73504	−1.24069	−2.21533	−2.11916	−0.92235	−0.89067
33	10.96948	−1.25656	−2.4134	−2.64925	−0.79452	−1.05998
34	11.90095	−1.17015	−2.77788	−2.58199	−0.96862	−1.02144
35	14.205	−1.00282	−3.98485	−2.30801	−1.39645	−1.13902
36	16.25321	−1.32547	−5.09509	−1.92308	−1.49429	−1.1651
37	17.96625	−1.26082	−5.81489	−1.80906	−1.58952	−1.17977
38	21.23983	−1.45496	−7.92596	−2.74174	−2.78922	−1.69821
39	20.05666	−0.77249	−7.04782	−2.45994	−3.22553	−1.49539
40	20.53598	−1.6985	−7.86833	−2.90659	−3.11132	−1.76431
41	18.50458	−1.78084	−7.00401	−3.02656	−2.86995	−1.69589
42	20.75746	−2.45329	−8.7462	−3.9763	−3.60375	−2.04416
43	22.11995	−3.68579	−10.1314	−4.76632	−3.60847	−2.54272
44	21.24168	−3.87245	−9.54882	−4.79398	−2.61987	−2.34513
45	24.90325	−4.87904	−12.0149	−5.38793	−2.91229	−2.86972
46	26.12748	−4.51018	−12.4606	−6.0043	−3.12754	−3.15355
47	27.92561	−4.89428	−13.5642	−6.76035	−2.95099	−3.36303
48	25.87956	−4.11228	−12.1302	−7.2883	−2.38254	−3.38384
49	28.83877	−3.32843	−13.5181	−7.61842	−4.28075	−3.81955
50	33.13329	−3.36866	−15.8124	−8.71257	−5.21303	−4.3486

**Table 2:** Vibration characteristics of degradation stages.

Degradation Stage	Physical Mechanism	Vibration Signature (Signs)
Early (Origins)	Small-pinpoint defects (pitting)	Low-amplitude pulses, high-frequency Kurtosis, Crest Factor.
Average	Expansion of defects, vibration	Increase in amplitude at characteristic defect frequencies
Late	General wear and tear, spread of defect	Increase in the overall root mean square value, modulation of the carrier frequency, noise background increases.

**Table 3:** Numerical values of approximation coefficients, errors and sensitivity parameters for the first six principal components.

Indicators	PCA_1_	PCA_2_	PCA_3_	PCA_4_	PCA_5_	PCA_6_
exponential approximation coefficients						
*a*	−8.0160	0.1521	0.5575	0.1608	0.1389	0.1675
*b*	0.0434	0.0819	0.1009	0.0988	0.0858	0.0908
*c*	4.6060	−0.0833	−0.1204	−0.0648	−0.0659	−0.0481
exponential approximation errors						
*RMSE*	1.8390	1.0420	2.2850	3.0490	0.4397	0.3741
*R*-square	0.9702	0.5223	0.7893	0.5990	0.9084	0.9092
sensitivity (steepness of the tangent in the pre-destruction area)						
*k*	1.4122	0.2729	1.1387	0.5467	0.2691	0.2605
*α*	54.6970	15.2690	48.7100	28.6690	15.0620	14.6030

## Data Availability

The original contributions presented in this study are included in the article. Further inquiries can be directed to the corresponding authors.

## References

[1] Lus T. (2015). The Valve Gear Systems Timing Parameters Identification for Marine Diesel Engines Diagnostics. J. Konbin.

[2] Aloisio A., Di Battista L., Alaggio R., Fragiacomo M. (2020). Sensitivity analysis of subspace-based damage indicators under changes in ambient excitation covariance, severity and location of damage. Eng. Struct..

[3] Banerjee S., Saravanan T.J. (2025). Robust data-driven online learning algorithm for precise structural damage localization using stochastic subspace identification. Measurement.

[4] Perederyi V., Nuzhniy S., Lebedenko Y. (2024). Diagnostics of the state and prediction of the residual resource of parts in extreme operating conditions. Adv. Mech. Eng. Transp..

[5] Sharko O., Buketov A., Klevtsov K., Sapronov O., Akimov O. (2024). Entropy model for determining the necessary information in the diagnostics of maritime transportation. Sci. J. TNTU.

[6] Sharko O., Yanenko A. (2023). Modeling intelligent software for the diagnostic and monitoring of ship power plant components using Markov chain. Sci.-Intensive Technol..

[7] Noman K., Li Y., Wen G., Patwari A.U., Wang S. (2024). Continuous monitoring of rolling element bearing health by nonlinear weighted squared envelope-based fuzzy entropy. Struct. Health Monit..

[8] Merkisz-Guranowska A., Waligyrski M. (2016). Analysis of vibroacoustic estimators for a heavy-duty diesel engine used in sea transport in the aspect of diagnostics of its environmental impact. J. Vibroeng..

[9] Burda E.A., Zusman G.V., Kudryavtseva I.S., Naumenko A.P. (2022). An Overview of Vibration Analysis Techniques for the Fault Diagnostics of Rolling Bearings in Machinery. Shock Vib..

[10] Liu L., Chen H., Li Z., Li W.-P., Liang Y., Feng H.-T., Zhou C.-G. (2022). A New Prediction Method for the Preload Drag Force of Linear Motion Rolling Bearing. Metals.

[11] Duan Y., Cao X., Zhao J., Li M., Yang X. (2023). A Spatiotemporal Fusion Autoencoder-Based Health Indicator Automatic Construction Method for Rotating Machinery Considering Vibration Signal Expression. IEEE Sens. J..

[12] Wang H., Ni G., Chen J., Qu J. (2020). Research on rolling bearing state health monitoring and life prediction based on PCA and Internet of things with multi-sensor. Meas. J. Int. Meas. Confed..

[13] Zinchenko S., Tovstokoryi O., Mateichuk V., Nosov P., Popovych I., Perederyi V. Automatic Prevention of the Vessel’s Parametric Rolling on the Wave. Proceedings of the COLINS-2024: 8th International Conference on Computational Linguistics and Intelligent Systems.

[14] Zinchenko S., Kyrychenko K., Grosheva O., Nosov P., Popovych I., Mamenko P. Automatic reset of kinetic energy in case of inevitable collision of ships. Proceedings of the 2023 13th International Conference on Advanced Computer Information Technologies (ACIT).

[15] Sharko M., Gonchar O., Tkach M., Polishchuk A., Vasylenko N., Mosin M., Petrushenko N. (2022). Intellectual Information Technologies of the Resources Management in Conditions of Unstable External Environment. Lecture Notes on Data Engineering and Communication Technologies.

[16] Sharko M., Petrushenko N., Gonchar O., Vasylenko N., Vorobyova K., Zakryzhevska I. Information Support of Intelligent Decision Support Systems for Managing Complex Organizational and Technical Objects Based on Markov Chains. Proceedings of the COLINS-2022: 6th International Conference on Computational Linguistics and Intelligent Systems.

[17] Sharko M., Gusarina N., Petrushenko N. (2019). Information-entropy model of making management decisions in the economic development of the enterprises. Adv. Intell. Syst. Comput..

[18] Sharko O., Stepanchikov D., Sharko A., Yanenko A. (2024). Computer Diagnostics of the Condition of Ship Rolling Bearings During Their Operation. Sci.-Based Technol..

[19] Louda P., Sharko A., Stepanchikov D., Sharko A. (2022). Experimental and Theoretical Study of Plastic Deformation of Epoxy Coatings on Metal Substrates Using the Acoustic Emission Method. Materials.

[20] Sharko A., Louda P., Nguyen V., Buczkowska K., Stepanchikov D., Ercoli R., Kascak P., Le V. (2023). Multicriteria Assessment for Calculating the Optimal Content of Calcium-Rich Fly Ash in Metakaolin-Based Geopolymers. Ceramics.

[21] Sharko A., Sharko O., Stepanchikov D., Ercoli R., Nguyen T.X., Tran D.H., Buczkowska K.E., Dancova P., Łos P., Louda P. (2023). Multi-criteria optimization of geopolymer foam composition. J. Mater. Res. Technol..

